# Devil's Work

**Published:** 1918-06-01

**Authors:** 


### The Hospital

The Workers' Newspaper of Administrative Medicine and Institutional Life, Administration, National Insurance and Health.

No. 1669, Vol. LXIV. SATURDAY, JUNE 1, 1918. PRICE ONE PENNY

### DEVIL'S WORK.

Well outside the battle area in the West of France is Etaples, where a constantly increasing number of hospitals of all kinds and for many purposes have been erected, with accommodation for many thousands of patients. A few of the hospitals may consist of old but substantial buildings, but in nearly every case they are hut or tent hospitals, with no sort of attempt or pretence to be anything but temporary, and quite incapable of resisting attack or saving their inmates from the effects of an air raid. The character and nature of the territory where these hospitals are situated is made abundantly clear by all kinds of devices, so that it is impossible for any airman to mistake the nature of the camps or the character of their inmates. Further than this, airmen are constantly flying over the district, and there cannot have been a single German airman on the Western Front who was not perfectly familiar with the Etaples hospitals, who did not fully appreciate that they were under the Red Cross flag and protection, and so sacred ground for all fighting men who had any manliness or courage or self-respect, let alone feelings of humanity and a recognition of the claims which the sick and injured have upon all hale members of mankind in war, as in peace.

When we spent a considerable time in Etaples and made ourselves familiar with the nature and character of the work, the condition and arrangements of the buildings, and the character of the various types of hospitals, the majority of which we personally inspected, we were struck with the fact that there was no attempt to make any clear division between any single hospital and its neighbours on all sides of it. The space between each hospital's site was simply a space of ground, seldom more than 2 feet in width, if so much, and it was no uncommon experience for all types of people engaged in the hospitals to lose themselves in trying to make an adjacent hospital for the first time. When the former raid was made by German aeroplanes on this hospital area there were some miraculous escapes, owing to a number of the shells just missing a tent or hut or burrowing beneath it and not exploding. The fact that there was a former raid adds to the heinous character of the outrage perpetrated by two squadrons of German Gothas, which flew over the camp on Whit Sunday night, the 19th ultimo, soon after 10 p.m., and continued their hellish work till after midnight. The *Times* reports that over a score of machines in two separate parties were employed, which dropped a great number of bombs, many of them large in size, making craters in the ground 15 and 20 feet across. It would be difficult to imagine any deliberately planned horror, invented by Satan himself, which could be more utterly devoid of every quality which civilised man, and even the most barbarous savages, have recognised should characterise the acts of any member of the human family, where sick, helpless, and dying men and defenceless men and women are concerned.

But the acts of these Germans were more than devilish, monstrous, cowardly, and self-condemning. We have indicated the slight character of the huts and tents, the absence of every kind of protection and defence or shelter for their inmates. They contained the patients' beds, which were filled with the helpless wounded. Some of them inside presented the appearance of a forest of trees, with the patients with their fractures accurately adjusted and fixed in frames of aluminium, suspended in the air by a system of pullies, in such a manner that they can move their bodies without disturbance of the injured limbs, the open wounds of which are at all times freely accessible. These cases had to be dressed, too, with infinite devotion and care, in the position in which they might be suspended. It would be well if every man, woman, and child throughout the civilised world could have the picture of the inside of one of these hospital huts or tents constantly before them. This should be accompanied by another picture showing the German airmen in the Gothas first exhausting their stock of heavy bombs, which they were dropping so that they might land somewhere among the attendants' quarters and the tents where the nursing sisters were moving amongst the rows of beds with their helpless occupants. A third picture might exhibit the next act of the enemy—i.e. the machines being brought down low enough to enable these air fiends to use their machine guns to rake the hospitals and the attendants' quarters with their fire. Of course, the mortality and terrible injuries inflicted upon already grievously wounded sufferers in the tent and hut hospitals, and upon the nursing sisters, orderlies, and doctors by machine guns, so deliberately aimed at such close quarters, can be better imagined than described. The result, no doubt, was, as the perpetrators of this deed, their masters, and superior officers desired it should be, a holocaust of slaughtered humanity, exhibiting a cowardice and concentrated abomination of criminal actions and intent which had never been attempted, much less exceeded, in the whole history of the world, prior to its perpetration. The simple truth is, and every civilised nation throughout the world should recognise it, that the hospitals attacked were well known to the enemy and their airmen who carried out this foul butchery of defenceless men and women, for very many wounded German prisoners have been treated there, since the war began, and were quite familiar with the nature of Etaples, its buildings, and the purposes to which they were put.

As we were preparing this article a striking piece of evidence of the truth of what is here urged was brought to us by a great hospital official who had come from the bedside of one of the patients who had occupied a bed at Etaples on that fatal Sunday evening. There were vacant beds in his ward, and one of them was soon occupied by the captain of the German Gotha brought down, or his colleague who escaped death with him but was injured. In the haughtiest possible way this man insisted upon the officer in command being sent for. When he arrived the German demanded that he should be at once transferred to England, on the ground that he was a combatant, and under the regulations he could not be retained in the centre of a war area, which Etaples was, as the raid that night had shown, and other raids would prove it to be in the near future. When he persisted he was informed that not only would he not be removed, but if other raids were coming, or in any case, he would be the very last living man to be moved from his bed. Mr. Bonar Law on Tuesday in the House of Commons stated that the raid caused over 300 casualties to hospital cases, and that a full report has been asked for and is expected. We fear that, when the casualties amongst doctors, sisters, nurses, orderlies, and the whole of the occupants of the hospitals affected are known, they will be found to be far greater in numbers, than Mr. Bonar Law appears to have any idea of.

It is no use wasting words in abusing Germans, whom this war has exhibited as being beyond the pale of the human family. They are a species apart, who have proved, with the approval and applause of their women and citizens, as well as their military rulers, that they hold nothing sacred, living or dead. The sooner the Allies, the British Government, we Britons, and All Associated With Us In This War Recognise The Facts, and deal with these foul and abandoned creatures in the only way which will protect humanity, effectually, and save the world from their domination, the juster will be our acts and conduct towards these enemies of ours, and the speedier will their complete isolation and adequate punishment be made as certain, as that night follows day.

